# Correlation of miRNAs with infarct volume in patients with acute ischemic stroke: A systematic review

**DOI:** 10.1097/MD.0000000000040728

**Published:** 2024-12-13

**Authors:** Yanmeng Yang, Guangming Wang, Yanlong Tang

**Affiliations:** a Radiology Department of the First Affiliated Hosptial of Dali University, Dali, Yunnan, China; b Clinical Medical Laboratory Centre, The First Affiliated Hospital of Dali University, Dali, Yunnan, China.

**Keywords:** infarct volume, miRNA, stroke, systematic review

## Abstract

**Background:**

Rapid diagnosis of acute ischemic stroke (AIS) remains challenging, and reliable biomarkers are needed. MicroRNAs (miRNAs) are endogenous small noncoding regulatory RNA molecules present in the serum, plasma, and saliva. miRNAs are considered to be sensitive biomarkers of tissue damage because of their high stability and relative tissue specificity. The aim of this systematic review was to assess the correlation between miRNAs and infarct volume in patients with AIS, to provide a basis for predicting ischemic stroke onset and improving prognosis in the clinic, among others.

**Methods:**

From the search of published Chinese and English literature in PubMed, Cochrane Library, CNKI, VIP, and Wanfang databases up to July 1, 2023, we performed a descriptive summary of the included studies. And use of 12 quality evaluation items and Cochrane Risk of Bias tool to assess the quality of included studies.

**Results:**

This systematic review included 17 studies with articles involving 1443 cases were included in the data extraction for a total of 18 miRNA indicators. Among them, 9 indicators were positively associated with infarct volume in patients with AIS, including endothelial microvesicles-miR-155, miR-146b, miR-181c, miR-182, miR-34a, miR-92a, miR-122-5p, miR-451a, and miR-409-3P.

**Conclusions:**

This study shows that miRNA can be used as a biomarker for AIS, reflecting the severity of neurological deficits in this patient and providing a basis for clinical judgement of the patient’s prognosis.

## 1. Introduction

Acute ischemic stroke (AIS) is a functional disorder of cerebral blood circulation that induces local softening or necrosis of brain tissue, cardiovascular disease, impaired consciousness, coma, etc, which is a common clinical symptom, with a high incidence rate, rapid progression of the disease and poor prognosis, which threatens patients’ lives and safety.^[1,2]^ It has been found that early detection and timely symptomatic treatment of ischemic stroke can effectively save brain tissues damaged by hypoxia and ischemia, increase neuronal synthesis and secretion, restore patients’ neurological function, and improve the prognosis, but there is no effective indicator for clinical prediction of the disease.^[3]^

Early imaging is difficult to detect significant lesions in AIS and repeated imaging is a financial burden for patients. In addition, the severity of neurological deficit is assessed using the modified Edinburgh-Scandinavia Score, National Institutes of Health Stroke Scale, Glasgow Oscillator Scale, and modified Rankin Scale, which are widely used but subjective, and are more reliable than the objective serological indicators and infarct volume measured. The latter is more reliable than objective serological indicators and infarct volume obtained by measurement.

Serological markers have become common clinical markers of choice for screening diseases due to their ease of sampling, low cost of testing and timeliness, and are also considered to be the most valuable adjunct to routine clinical examination and imaging data.^[4]^ Over the past decade or so, skillful progress has been made in understanding the pathophysiology and biochemistry of AIS, and some studies have identified a number of ischemic injury-associated proteins, such as interleukin-6, matrix metalloproteinase-9, C-reactive protein, calcium-binding protein B (S100B), neuron-specific enolase, myelin basic protein, and glial fibrillary acidic protein biomarkers as tools to aid in the diagnosis of ischemic stroke, but their specificity and ability to differentiate between AIS and its associated risk factors have significant limitations.^[5,6]^

MicroRNAs (miRNAs) are endogenous small noncoding regulatory RNA molecules that interact with target coding messenger RNAs to inhibit translation or degrade messenger RNA transcripts. miRNAs are present in all types of body fluids, including serum, plasma, and saliva, and are considered to be sensitive biomarkers of tissue damage due to their high stability, relative tissue specificity, relevance to disease states, easily detectable concentrations, ease of manipulation, and measurement sensitivity, miRNAs are considered sensitive biomarkers of tissue damage.^[7]^ miRNAs are closely associated with the pathogenesis of AIS, including effects on neuronal cell survival, dysregulation of neurovascular integrity, and AIS-mediated inflammation.^[8]^ Blood mRNA gene expression profiles can be used as genomic biomarkers of AIS and even as features of AIS subtypes.^[9,10]^ Exploring the correlation between miRNA and infarct volume can more accurately assess the severity and determine the prognosis of patients with ischemic stroke.

## 2. Method

### 2.1. Literature search

A comprehensive literature search was conducted on 6 online databases (PubMed, Cochrane Library, CNKI, WEIPU, WANGFANG, and YALU) to identify relevant articles, from inception to June 1, 2023. The following search terms were used: “MicroRNA” or “miRNAs” or “Micro RNA” or “miRNA” and “MicroRNA.” or “miRNAs” or “Micro RNA” or “miRNA” and “ Stroke Volume” or “Stroke Volumes” or “infarct volume” or “ Volume, Stroke.” The 2 authors double-checked the retrieved titles and abstracts and resolved any differences that arose through discussion.

### 2.2. Study selection criteria

The following criteria had to be met for inclusion: (1) studies exploring the correlation between miRNAs and infarct volume in patients with AIS; (2) studies comparing miRNA expression in patients with AIS with similar age- and sex-matched healthy controls; and (3) review articles and articles from animal studies were excluded from the study.

The criteria for inclusion of patients were (1) confirmation of AIS by magnetic resonance imaging or computed tomography head scan. (2) Collection of a biospecimen within 72 hours of the onset of AIS and before receiving any treatment. Patients and healthy controls were excluded if they had: (1) suffered from other diseases, including acute infections, immune disorders, neurodegenerative diseases, and cancers. (2) Taken medications such as low molecular heparin or normal heparin within the last month. (3) Suffered from secondary cerebral hemorrhage.

### 2.3. Data extraction and quality assessment

Information was obtained from the original and supplementary documents studied, Two reviewers independently performed screening, data extraction, and all included studies were assessed according to Revman and 12 quality evaluation items^[11]^: report of source information, report of inclusion criteria, report of exclusion criteria, report of the time frame of recruitment, report of recruitment setting, subjects consecutively recruited, validated questionnaire (patients were all analyzed, control or assessment of confounding, report of missing data, missing data imputed, and report of response rate). Each item was scored “1” if the criterion item was met, and “0” if the criterion item was not met. Two reviewers independently assessed the quality of the included studies. In a consensus meeting the scores were compared. When there was disagreement, a consensus was reached by means of discussion. In case of persistent disagreement, a third reviewer gave the final judgment.

## 3. Result

### 3.1. Characteristics of included studies

**Figure 1.**
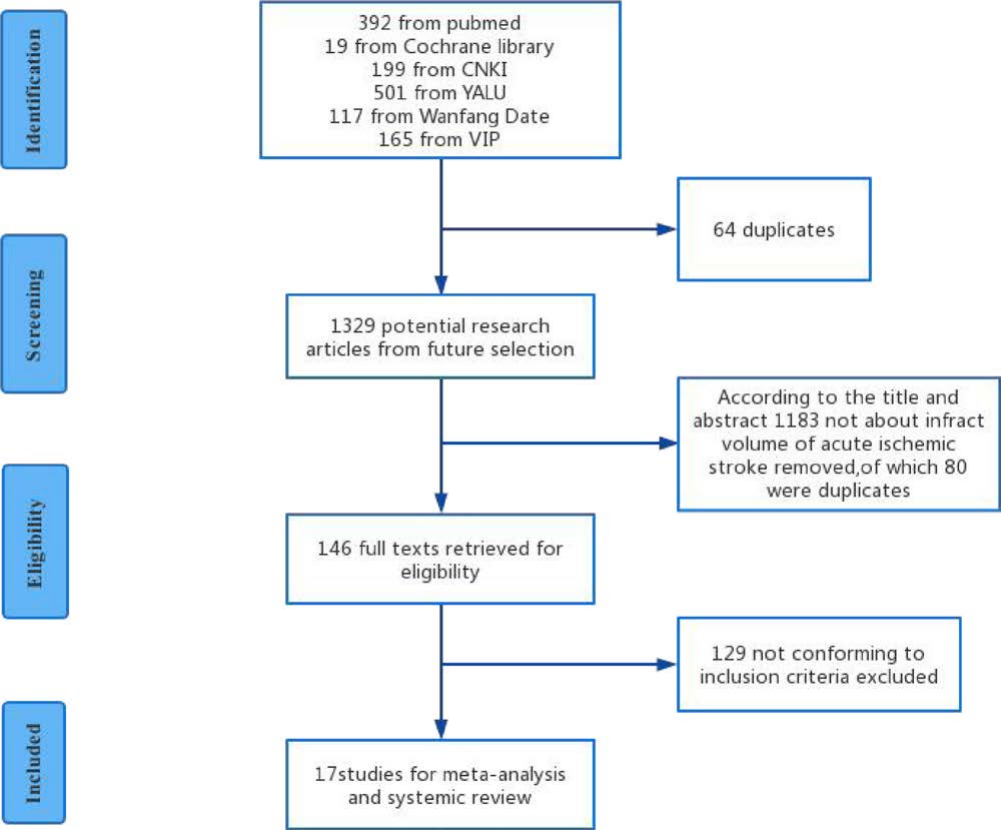
Flow diagram of literature inclusion. Identification, screening, eligibility extraction, and inclusion steps of studies were depicted.

Figure 1 shows a flowchart of the literature search and study selection, with a total of 17 studies with 18 miRNA indicators included and further assessed using the Cochrane Risk of Bias tool. Table 1 summarizes the basic characteristics of the studies.

**Table 1:** General information of the included studies.

Article	AIS			Control			Infact volume	Time	Type	Tech	microRNA	r	*P*	Correlations
	N	Age	Sex (M)	N	Age	Sex (M)								
Peng Zhang (2021)^[12]^	92	NA	NA	68	69.6 ± 5.8	NA	Diameter	Within 24 hours	Serum	NA	miR-181c	NA	*P* = .000	Positive
											miR-146a	NA	*P* = .001	Negative
Shiliang Yang (2023)^[13]^	80	59.24 ± 6.61	51	80	57.49 ± 8.74	45	Pullcino	Within 24 hours	Serum	qRT-PCR	miR-181c	*R* = 0.752	*P* < .05	Positive
											miR-92a	*R* = 0.832	*P* < .05	Positive
											miR-34a	*R* = 0.806	*P* < .05	Positive
Huiting Zhang (2020)	93	67. 5 ± 11.2	57	70	67. 5 ± 11.2	39	NA	NA	NA	NA	EMVs-miR-155	NA	NA	Positive
Jinli Feng (2018)^[14]^	90	58.9 ± 10.3	57	40	56.3 ± 9.4	23	Pullcino	Within 24 hours	Plasma	qRT-PCR	miR-124	*r* = -0.577	NA	Negative
Song Li (2016)	22	NA	NA	NA	NA	NA	DWI	Within 24 hours	Serum	qRT-PCR	miR-124	NA	NA	Negative
											miR-9	NA	NA	Negative
											miR-219	NA	NA	Negative
Zunchun Xie (2019)^[15]^	40	65.03 ± 10.18	28	40	63.18 ± 8.16	25	DWI	Within 24 hours	Plasma	qRT-PCR	miR-124	*r* = -0.473	*P* < .05	Negative
Qiuhong Ji (2016)^[16]^	65	64 (54–70)	40	66	60 (53–64)	36	DWI	Within 24 hours	Serum	qRT-PCR	miR-124	*R* = 0.6312	*P* < .01	Positive
											miR-9	*R* = 0.6768	*P* < .01	Positive
Nanyao Chen (2019)	120	61.0 ± 6.7	71	80	62. 0 ± 6. 5	42	Pullcino	Within 24 hours	Serum	qRT-PCR	miR-124	*r* = -0.613	*P* < 0. 01	Negative
											miR-182	*R* = 0. 761	*P* < 0. 01	Positive
Zhenzhen Chen (2018)^[4]^	128	68.42 ± 17.26	109	102	65.36 ± 16.32	74	DWI	Within 24 hours	Serum	qRT-PCR	MiR-146b	*R* = 0.5199	*P* < .0001	Positive
Yan Jiang (2022)	102	65.52 ± 2.17	58	102	65.52 ± 2.17	58	NA	Within 24 hours	Serum	qRT-PCR	miR-151a-3p	NA	*P* < .05	Positive
											miR-210	NA	*P* < .05	Negative
Yanping Liu (2014)^[17]^	31	66.32	21	11	NA	NA	DWI	Within 24 hours	Serum	qRT-PCR	miR-214	*r* = -0.423	*P* = .022	Negative
											miR-9	*r* = -0.608	*P* < .001	Negative
Yajing Chen (2017)^[18]^	50	64 ± 9.4	32	33	63 ± 7.9	20	Pullcino	Within 24 hours	Serum	qRT-PCR	miR-223	*R* = 0.20	*P* = .19	NA
Wenwen Pei (2023)	128	58.62 ± 10.05	71	128	58.14 ± 9.37	75	CT	Within 24 hours	Serum	qRT-PCR	miR-26b	*r* = -0.593	*P* < .001	Negative
Linlin Liu (2023)	152	60.23 (49–71)	85	60	60.09 (47–71)	39	Pullcino	Within 24 hours	Serum	qRT-PCR	miR-34a	*R* = 0.788	*P* = .000	Positive
Ting-Ying Liang (2016)^[19]^	102	65.1 ± 10.0	69	97	6.52 (51~68)	67	Pullcino	Within 24 hours	Serum	qRT-PCR	miR-34a-5p	*r* = -0.719	*P* > .05	Negative
Le Wang (2023)	120	55.6 ± 14.6	76	80	56.2 ± 18.5	53	Pullcino	Within 24 hours	Serum	qRT-PCR	miR-409-3P	NA	*P* < .05	Positive
Ying Kong (2020)^[20]^	28	NA	NA	18	NA	NA	NA	Within 24 hours	Serum	qRT-PCR	miRNA-122-5p	NA	NA	Positive
											miRNA-451a	NA	NA	Positive

### 3.2. Quality assessment of the included studies

**Figure 2.**
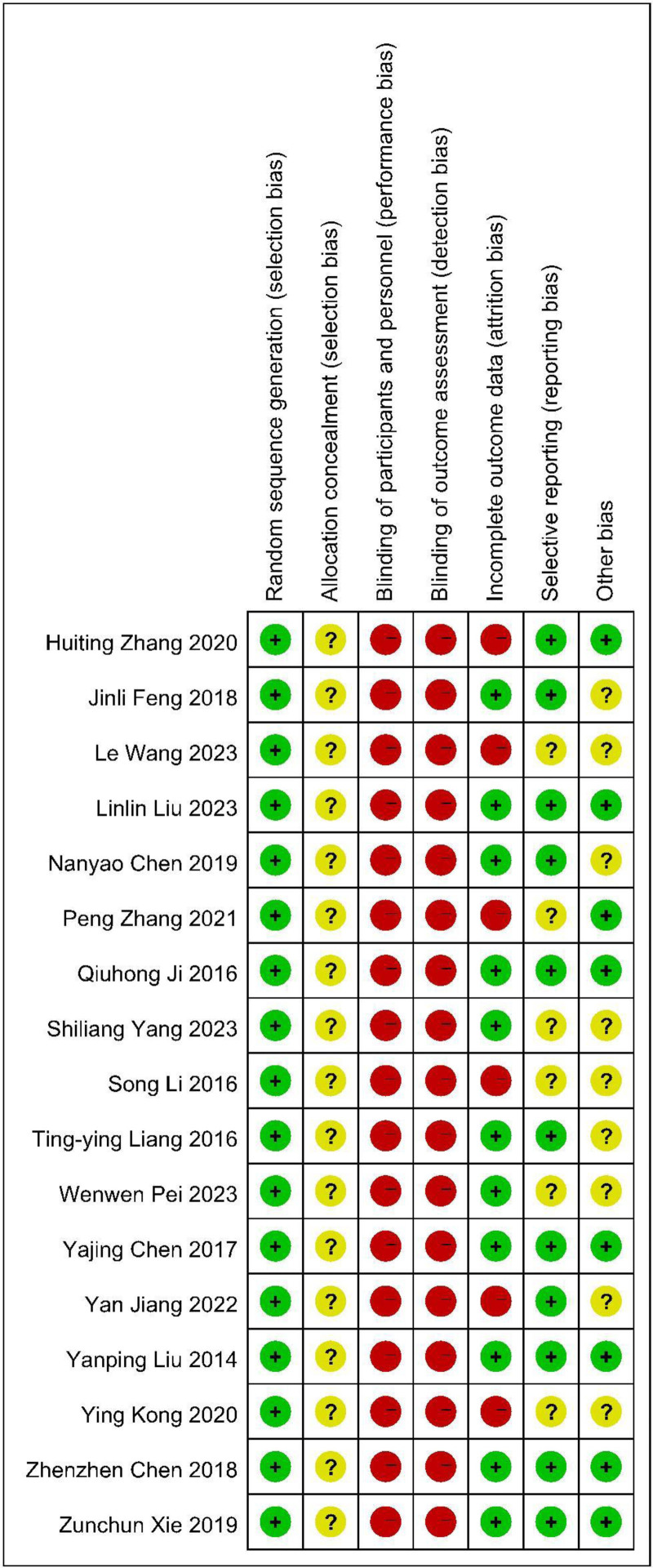
Risk of bias assessment. Green: low risk, red: high risk, and yellow: unclear levels of risk.

The quality of the included studies was good. From the 17 methodological items assessed in the quality assessment, 9 studies had a score of 7 out of 17. But validation of questionnaires, whether all patients were analyzed, whether confounding has been assessed or controlled for, and if missing data was imputed were hardly reported (Table 2). Assessments of low risk are shown in green, assessments of high risk are shown in red, and unclear levels of risk are shown in yellow (Fig. 2).

**Table 2:** Study quality assessment.

Quality item study	1	2	3	4	5	6	7	8	9	10	11	12	Sum
Peng Zhang (2021)^[12]^	+	+	+	+	+	-	-	+	-	-	-	-	6
Nanyao Chen (2019)	+	+	+	+	+	-	-	+	-	-	-	+	7
Shiliang Yang (2023)^[13]^	+	+	+	+	+	-	-	+	-	-	-	+	7
Yan Jiang (2022)	+	+	+	+	+	-	-	+	-	-	-	-	6
Wenwen Pei (2023)	+	+	+	+	+	-	-	+	-	-	-	+	7
Le Wang (2023)	+	+	+	+	+	-	-	+	-	-	-	+	7
Linlin Liu (2023)	+	+	+	+	+	-	-	+	-	-	-	+	7
Jinli Feng (2018)^[14]^	+	+	+	+	+	-	-	+	-	-	-	+	7
Song Li (2016)	+	+	?	-	+	-	-	+	-	-	-	-	4
Zunchun Xie (2019)^[15]^	+	+	?	-	+	-	-	+	-	-	-	+	5
Zhenzhen Chen (2018)^[4]^	+	+	+	+	+	-	-	+	-	-	-	+	7
Huiting Zhang (2020)	+	+	+	+	+	-	-	+	-	-	-	-	6
Yanping Liu (2014)^[17]^	+	+	+	+	+	-	-	+	-	-	-	-	6
Ying Kong (2020)^[20]^	+	+	?	-	+	-	-	+	-	-	-	-	4
Ting-Ying Liang (2016)^[19]^	+	+	+	+	+	-	-	+	-	-	-	+	7
Yajing Chen (2017)^[18]^	+	+	+	+	+	-	-	+	-	-	-	+	7
Qiuhong Ji (2016)^[16]^	+	+	?	+	+	-	-	+	-	-	-	+	6
Sum	17	17	13	14	17	0	0	17	0	0	0	11	

*Question*s: (1) Is the source of information reported? (2) Were inclusion criteria reported? (3) Were exclusion criteria reported? (4) Was the time frame of recruitment reported? (5) Was the recruitment setting reported? (6) Were subjects consecutively recruited? (7) Has the questionnaire been tested for measurement properties? (8) Have all patients been analyzed? (9) Has confounding been assessed and controlled for? (10) Were missing data reported? (11) Were missing data imputed? (12) Was response rate reported?

− = No, ? = not described, + = Yes, NA = not applicable.

### 3.3. Positive

The ones that were clearly positively correlated with infarct volume included endothelial microvesicles (EMVs)-miR-155, miR-146b, miR-181c, miR-182, miR-34a, miR-92a, miR-122-5p, miR-451a, and miR-409-3P for a total of 9 indicators.

The study by Zhang et al suggests that EMVs-miR-155 may contribute to the severity of ischemic stroke (IS) by regulating oxidative stress and apoptosis. Because EMVs can reflect the status of endothelial cells (EC) involved in the pathogenesis of IS. miR-155 can regulate EC function. miR-155 is a major carrier of circulating miRNAs^[21]^, MVs protect miRNAs from circulating ribonuclease (RNase) and stabilize them in the circulation.^[22]^ miRNAs in EMVs are involved in EC dysfunction under pathological conditions and may serve as more specific and stable biomarkers of vascular disease. miR-155 is a multifunctional miRNA that regulates a wide range of physiological and pathological processes, and down-regulation of miR-155 reversed the inhibitory effect of inflammatory cytokines on eNOS expression in human umbilical vein endothelial cells.^[23,24]^ Excessive oxidative stress and apoptosis have been shown to trigger the release of EMV which in turn promotes EC dysfunction.^[25]^ In the TOAST classification, it was shown that antioxidants were significantly lower in the large artery atherosclerosis group of IS patients than in small vessel disease,^[26]^ and that levels of oxidized LDL were significantly higher in large artery atherosclerosis compared to small vessel disease.^[27]^ Therefore, it can be suggested that EMVs-miR-155 may contribute to the severity of IS by regulating oxidative stress and apoptosis.

The studies of Peng Zhang^[12]^ and Shiliang Yang^[13]^ concluded that miR-181c was positively correlated with infarct volume. miR-181c is involved in mitochondrial genomic protein coding, which affects mitochondrial function. miR-181c levels are abnormal, resulting in down-regulated expression of the mt-cox1 protein and upregulation of mt-cox2 and mt-cox3 proteins, which leads to mitochondrial complex IV remodeling and mitochondrial dysfunction. Therefore, it is believed that reducing the expression of miR-181c can reduce neuronal damage.

Some studies^[28]^ have confirmed that miR-182 can promote apoptosis and aggravate brain injury by down-regulating the expression of antiapoptotic proteins. miR-182 can also be involved in the regulation of oxidative stress by promoting the production of free radicals and decreasing the activity of antioxidants, which further aggravates brain injury, and it is closely associated with the onset of ischemic stroke and the deterioration of the condition of brain injury. It can be speculated that miR-182 may be involved in the whole pathophysiological process of the disease, and its specific mechanism of influence may involve the regulation of inflammation and apoptosis, which also has an important influence on the prognosis of patients.

The study of Yang Shiliang et al concluded that the higher the serum miR-92a and miR-34a levels, the worse the prognosis of AIS patients. Consistent with the findings of Liu Linlin et al serum miR-92a can negatively regulate angiogenesis after cerebral ischemia and can promote inflammation progression. miR-92a can promote the progression of atherosclerosis and the formation of atherosclerotic plaques when the level of miR-92a is elevated, which can increase the degree of ischemic stroke and aggravate the damage of brain tissue.^[29]^ miR-34a is widely expressed in the whole body tissues, such as the brain, cardiomyopathies, lungs, livers and other tissues, and is involved in various biological processes, such as angiogenesis, cell apoptosis miR-34a is widely expressed in brain.^[30,31]^

Ying Kong et al showed that the number of peripheral blood natural killer (NK) cells was reduced and miR-451a and miR-122-5p were significantly upregulated in NK cells in patients with AIS. miR-451a and miR-122-5p were suggested to possibly contribute to the impaired NK cell-mediated immune defenses after AIS, which was positively correlated with the severity of neurological deficit positively correlated with the severity of neurological deficits. NK cells are cytotoxic innate lymphocytes that are critical for early host defense against pathogens.^[32]^ Investigators identified reduced human peripheral blood NK cell counts, cytokine production, and cytotoxicity in human NK cells obtained from patients with AIS. The degree of loss of NK cell numbers and activity correlated with infarct volume. miRNA sequencing analysis revealed that cerebral ischemia significantly altered the expression profile of miRNAs in circulating NK cells. Importantly, inhibition of miR-451a or miR-122-5p enhanced the expression of activation-related receptors in NK cells.^[20]^ It suggests that miRNAs play an important role in stroke-induced NK cell-mediated immune defense injury.

MiR-146b has been most studied in the proliferation, differentiation, and phenotypic transformation of vascular smooth muscle cells. In the cardiovascular system, miR-146b is not only important for cardiac and vascular development, but also plays an important role in cardiac pathogenic factors (e.g., hypertrophy and ischemia), which has been regarded as a new therapeutic target for vascular disease, and it was demonstrated that miR-146b has a very similar sensitivity and specificity to interleukin-6 in predicting AIS,^[4]^ so that it is thought that the upregulation of miR-146b predicts a higher severity of neurological deficits in AIS patients.

Wang Le et al concluded that miR-409-3P could inhibit the expression of CTRP3 in PC12 cells, and serum miR-409-3P was correlated with the severity of AIS, which was clinically significant as a molecular marker for the early diagnosis of AIS. It has been found that miR-409-3P can inhibit nasopharyngeal carcinoma development by negatively regulating the expression of RRM2,^[33]^ and can also regulate FABP4 in ovarian cancer cells and thus affect the metabolic pathways in ovarian cancer cells,^[34]^ and the analysis in AIS may be that miR-409-3P can target peroxisome proliferator-activated receptor γ to regulate the activation of microcellularity and inflammatory factor expression. miR-409-3P overexpression significantly increased inflammatory cytokine expression, promoting microglia activation and inflammatory cytokine expression, leading to further disease progression.

### 3.4. Negative

There were 6 indicators that were negatively correlated with infarct volume, including miR-146a, miR-151a-3p, miR-210, miR-219, miR-26b, and miR-34a-5p.

MiR-146a has a strong inhibitory effect on the proliferation and differentiation of vascular endothelial cells, mediates directional cell transfer, and affects the chemotaxis of lymphocytes, macrophages, and neutrophils in inflammatory responses. When serum miR-146a expression is down-regulated, the corresponding chemokine receptors on epithelial cells, neutrophils, endothelial cells, and lymphocytes are activated, leading to hyperfibrinolysis, which ultimately leads to increased blood coagulation and blood viscosity, contributing to the formation of thrombus and inducing AIS. The included studies showed that miR-146a was negatively correlated with infarct volume, and the upregulation of miR-146a was associated with a lower severity of AIS.^[12]^

MiR-151a-3p has the function of activating the oxidative stress response of the organism and can participate in the signaling of the nuclear factor-κB pathway, so its elevated level can aggravate the cerebral ischemia/reperfusion injury of the patients, and then damage the cerebral nerves.^[14]^ Serum miR-210 can sensitively regulate its own expression level in response to changes in the degree of hypoxia, and lower levels indicate that the degree of hypoxia in patients is more severe and brain damage is more serious.^[35]^ Zeng et al proposed miRNA-210, a major pleiotropic hypoxic miRNA, as a sensitive blood biomarker for clinical diagnosis and prognosis of acute cerebral ischemia, without exploring its relationship with infarct volume.^[36]^ Therefore, changes in AIS patients can be assessed by detecting serum miR-151a-3p, miR-210 and inflammatory factor levels.

MiR-26b is a hypoxia-regulated miRNA whose expression is down-regulated in hypoxic environments. miR-26b regulates platelet activation, vascular regeneration, and neurons, and down-regulation of megakaryocyte and platelet miR-26b contributes to elevated levels of platelet activation status in sepsis.^[37,38]^ Overexpression of miR-26b attenuates cognitive deficits by reducing damage to the CA1 region of the rat hippocampus, inhibiting microglia activation, preventing inflammatory factor production, and reducing neuronal apoptosis.^[39]^ It is suggested that low expression of miR-26b may increase platelet activation, promote inflammation and thrombosis, aggravate nerve injury, and lead to poorer prognosis of patients.

A study by Liang et al^[19]^ concluded that miR-34a-5p is mainly sensitive to mild stroke. This negative correlation also implied a potential neuroprotective role of miR-34a-5p in stroke. Welch et al^[40]^ demonstrated that increased expression of miR-34a-5p led to increased apoptosis, the mechanism of cysteine aspartate-dependent apoptosis in AIS. All these findings reveal important changes in miRNAs in ischemic stroke and suggest that miR-34a-5p may be a viable molecular predictor in the development of AIS.

Changes in miR-219 expression may alter inflammatory response transcripts and peripheral pro-inflammatory cytokines in the brain, thereby contributing to disruption of the blood–brain barrier.^[41]^ MiR-219 has been shown to reduce leukotriene production and to play an important role in the self-limiting resolution of acute inflammation.^[42,43]^

### 3.5. Correlation of miR-9 and miR-124 with infarct volume is controversial

Serum exosomes miR-9 and miR-124 are 2 brain-specific microRNAs that are promising biomarkers for the diagnosis of AIS and assessment of the extent of damage in ischemic injury.^[16,17]^ As well as miR-9 and miRNA-124 function in anti-inflammatory actions by inhibiting the production of pro-inflammatory cytokines.^[44]^ MiR-124 has been found to promote microglial cell quiescence and inhibit monocyte and macrophage activation in the central nervous system during experimental autoimmune encephalomyelitis^[45]^; overexpression of miR-9 has been found to inhibit interleukin-1β-induced tumor necrosis factor-a production and secretion of matrix metalloproteinase-13 in isolated human chondrocytes,^[46]^ and regulates the expression of nuclear factor jB1 in human monocytes and neutrophils exposed to pro-inflammatory signals, thereby preventing inappropriate inflammatory responses.^[44]^ Six studies in this inclusion examined the relationship between miR-124 and infarct volume. The study of Xie Zunchun et al,^[15]^ concluded that miR-124 was negatively correlated with infarct volume, and the results were consistent with the results of animal experiments,^[47]^ which showed a significant reduction in ischemic stroke area after intracranial injection of miR-124 mimetic liposomes in mice with a middle cerebral artery occlusion model, and concluded that miR-124 had a protective and ameliorative effect on neurological function in patients with AIS. And the study of Qiuhong Ji et al concluded that miR-124 and miR-9 in serum exosomes were upregulated in AIS patients and positively correlated with infarct volume. The results were also contrary to the findings of Yanping Liu et al.^[17]^

### 3.6. MiR-223 was not considered to be significantly correlated with infarct volume

miR-223 was shown to be one of the most highly expressed miRNAs in plasma exosomes of healthy individuals but it did not significantly correlate with infarct volume in ischemic stroke patients.^[18]^

## 4. Discussion

Cerebral infarction is a general term for ischemic stroke, and traditionally, the diagnosis of stroke has depended largely on examination by clinical caregivers and various neuronal imaging techniques. However, these diagnostic methods can only confirm a patient’s disease state, not predict the disease. Therefore, there is a great need for a reliable and easily detectable circulating biomarker to predict the risk and/or outcome of AIS. Patient-based studies have reported some changes in circulating miRNA expression during cerebral ischemia. And it has been shown that there are faster, more accurate, and cost-saving methods available for miRNA detection. Hertenstein et al^[48]^ showed that their customized device enables real-time monitoring of emulsion LAMP without the need to add additional probes/dyes in a time-efficient, cost-effective, and space-saving manner. The customized device enables rapid and sensitive detection of miRNA. The short sequence and high sequence similarity of miRNAs hindered the detection, so Zhong and his team^[49]^ propose a method to integrate polyA-tailing and CRISPR/Cas12a to amplify and detect all miRNAs with high specificity and sensitivity.

In addition, the literature contains evidence of miRNA expression patterns in the circulatory system of ischemic patients. For example, significant changes in circulating levels of miR-30a and miR-126 were observed, suggesting that miR-30a, miR-126 may be potential biomarkers for the diagnosis of ischemic stroke.^[50]^ Serum miRNA-221-3p and miRNA-382-5p may be used as potential noninvasive biomarkers for the diagnosis of ischemic stroke.^[51]^ MiR-21 and miR-221 are not specific for vascular disease may not be used as diagnostic markers.^[52]^ MiR15a and miR-16 are increased in serum from patients with severe limb ischemia,^[53]^ miR-15a has been identified as a key factor in ischemic stroke^[54]^, and miR-17-5p may be a factor in adult neurogenesis after stroke.^[55]^ MiR-19b, miR-29b-2, miR-339-5p are upregulated after ischemic injury in animal models.^[56]^ However, the correlation between the above indicators and the severity of neurological deficits in patients with AIS remains to be investigated.

There are many mechanisms by which miRNAs affect neurological deficits in AIS patients, mainly by promoting apoptosis and generating inflammatory responses to affect brain tissue damage such as miR-182; they can also be involved in mitochondrial genome protein coding, such as miR-181c, which affects the function of mitochondria, and increases neuronal damage; they can also be used as inducing factors that induce immune cell-mediated immune damage. It can also act as an inducing factor to induce immune cell-mediated immune damage, affecting neurological function; affecting coagulation function to promote thrombosis; and causing oxidative stress, which can lead to damage to the nervous system due to local inflammation caused by harmful factors entering the brain parenchyma through the blood–brain barrier, and so on.

In summary, miRNA has the potential to be used as a reliable biomarker for determining the prognosis of AIS patients. The correlation between miRNA and the severity of neurological deficits in AIS patients as judged by the relationship between miRNA and infarct volume is more objective and reliable than the previous methods, which can provide strong evidence for clinical judgement of the prognosis of AIS. However, the mechanism of various miRNAs requires in-depth research and discussion. The combination of serological indexes and imaging data to diagnose the disease has always been the main clinical diagnostic method, and both of them are objective facts, which improve the reliability and accuracy of the diagnosis and prediction of the disease.

## 5. Conclusions

This study shows that miRNA can be used as a biomarker for AIS, reflecting the severity of neurological deficits in this patient and providing a basis for clinical judgement of the patient’s prognosis.

## Author contributions

**Conceptualization:** Yanlong Tang.

**Methodology:** Guangming Wang.

**Writing – original draft:** Yanmeng Yang.

## References

[1] Jiang Q Xiao S Shu L Huang X Chen X Hong H. Pituitary apoplexy leading to cerebral infarction: a systematic review. Eur Neurol. 2020;83:121–30. 32544913 10.1159/000507190

[2] Fujino Y Kawasaki T Kawamata H Tamura A Shiga K Oyamada H. Cerebral infarction with pulmonary thromboembolism due to immobilization. Intern Med. 2020;59:2955–9. 32713906 10.2169/internalmedicine.3285-19 PMC7725618

[3] Takeda H Yamaguchi T Yano H Tanaka J. Microglial metabolic disturbances and neuroinflammation in cerebral infarction. J Pharmacol Sci. 2021;145:130–9. 33357771 10.1016/j.jphs.2020.11.007

[4] Chen Z Wang K Huang J. Upregulated serum MiR-146b serves as a biomarker for acute ischemic stroke. Cell Physiol Biochem. 2018;45:397–405. 29402769 10.1159/000486916

[5] Jickling GC Sharp FR. Blood biomarkers of ischemic stroke. Neurotherapeutics. 2011;8:349–60. 21671123 10.1007/s13311-011-0050-4 PMC3250275

[6] Ramos-Fernandez M Bellolio MF Stead LG. Matrix metalloproteinase-9 as a marker for acute ischemic stroke: a systematic. J Stroke Cerebrovasc Dis. 2011;20:47–54. 21044610 10.1016/j.jstrokecerebrovasdis.2009.10.008

[7] Laterza OF Lim L Garrett-Engele PW. Plasma MicroRNAs as sensitive and specific biomarkers of tissue injury. Clin Chem. 2009;55:1977–83. 19745058 10.1373/clinchem.2009.131797

[8] Tan JR Koo YX Kaur P. microRNAs in stroke pathogenesis. Curr Mol Med. 2011;11:76–92. 21342133 10.2174/156652411794859232

[9] Sharp FR Jickling GC Stamova B. RNA expression profiles from blood for the diagnosis of stroke and its causes. J Child Neurol. 2011;26:1131–6. 21636778 10.1177/0883073811408093 PMC3674558

[10] Jickling GC Xu H Stamova B. Signatures of cardioembolic and large-vessel ischemic stroke. Ann Neurol. 2010;68:681–92. 21031583 10.1002/ana.22187 PMC2967466

[11] Zeng X Zhang Y Kwong JS. The methodological quality assessment tools for preclinical and clinical studies. J Evid Based Med. 2015;81:2–10. 10.1111/jebm.12141 25594108

[12] Zhang P. Correlation analysis of serum miR-181c and miR-146a levels with lesion volume and neurological deficits in elderly patients with acute cerebral infarction. Med Theory Pract. 2022;3518:3173–5. (Chinese).

[13] Yang SL Xie ZM Du J. Serum microRNA-92a, microRNA-34a, and microRNA-181c levels are valuable in the diagnosis and prognostic assessment of acute cerebral infarction. J Acute Crit Care Intern Med. 2023;2901:41–5. (Chinese).

[14] Feng JL Wang W. Correlation of miR-124 expression with IL-19 and CRP in patients with acute cerebral infarction. J Brain Neurol Dis. 2018;2602:90–4. (Chinese).

[15] Xie ZC Liu B Zhou MH Chen YK. Changes in plasma miR-124 expression in patients with acute ischaemic stroke and its significance. J Pract Med. 2019;3503:343–5. (Chinese).

[16] Ji Q Ji Y Peng J. Increased brain-specific MiR-9 and MiR-124 in the serum exosomes of acute ischemic stroke patients. PLoS One. 2016;11:e0163645. 27661079 10.1371/journal.pone.0163645 PMC5035015

[17] Liu Y Zhang J Han R Liu H Sun D Liu X. Downregulation of serum brain specific microRNA is associated with inflammation. J Clin Neurosci. 2015;22:291–5. 25257664 10.1016/j.jocn.2014.05.042

[18] Chen Y Song Y Huang J. Increased circulating exosomal miRNA-223 is associated with acute ischemic stroke. Front Neurol. 2017;8:57. 28289400 10.3389/fneur.2017.00057 PMC5326773

[19] Liang TY Lou JY. Increased expression of mir-34a-5p and clinical association in acute ischemic. Med Sci Monit. 2016;22:2950–5. 27545688 10.12659/MSM.900237 PMC5004986

[20] Kong Y Li S Cheng X. Brain ischemia significantly alters microRNA expression in human peripheral blood. Front Immunol. 2020;11:759. 32477329 10.3389/fimmu.2020.00759 PMC7240012

[21] Kuhn S Splith K Ballschuh C. Mononuclear-cell-derived microparticles attenuate endothelial inflammation by transfer of miR-142-3p in a CD39 dependent manner. Purinergic Signal. 2018;14:423–32. 30244433 10.1007/s11302-018-9624-5 PMC6298922

[22] Boon RA Vickers KC. Intercellular transport of microRNAs. Arterioscl Throm Vas. 2013;33:186–92. 10.1161/ATVBAHA.112.300139 PMC3580056 23325475

[23] Lv J Yang L Guo R Shi Y Zhang Z Ye J. Ox-LDL-induced MicroRNA-155 promotes autophagy in human endothelial cells via repressing the Rheb/ mTOR Pathway. Cell Physiol Biochem. 2017;434:1436–48. 10.1159/000481875 29017169

[24] Sun HX Zeng DY Li RT. Essential role of microRNA-155 in regulating endothelium-dependent vasorelaxation by targeting endothelial nitric oxide synthase. Hypertension. 2012;60:1407–14. 23108656 10.1161/HYPERTENSIONAHA.112.197301

[25] Deng F Wang S Xu R Yu W Wang X Zhang L. Endothelial microvesicles in hypoxic hypoxia diseases. J Cell Mol Med. 2018;228:3708–18. 10.1111/jcmm.13671 PMC6050493 29808945

[26] Tsai NW Chang YT Huang CR Lin HF Lin RT Juo SH. Association between oxidative stress and outcome in different subtypes of acute ischemic stroke. Biomed Res Int. 2014;2014:256879. 24895559 10.1155/2014/256879 PMC4034452

[27] Wang A Yang Y Su Z. Association of oxidized low-density lipoprotein with prognosis of stroke and stroke subtypes. Stroke. 2017;48:91–7. 27899755 10.1161/STROKEAHA.116.014816

[28] Yi H Huang Y Yang F Liu W He S Hu X. MicroRNA-182 aggravates cerebral ischemia injury by targeting inhibitory member of the ASPP family (iASPP). Arch Biochem Biophys. 2017;620:52–8. 27242323 10.1016/j.abb.2016.05.002

[29] Cai XR Zhang XY Pan JX. Advances in the study of fibrinogen activator inhibitor-1. J Clin Pathol. 2020;4002:458–63. (Chinese).

[30] Vaitkiene P Pranckeviciene A Stakaitis R Steponaitis G Tamasauskas A Bunevicius A. Association of miR-34a expression with quality of life of glioblastoma patients: a prospective study. Cancers (Basel). 2019;11:300. 30836600 10.3390/cancers11030300 PMC6468714

[31] Chua CEL Tang BL. miR-34a in neurophysiology and neuropathology. J Mol Neurosci. 2019;67:235–46. 30488149 10.1007/s12031-018-1231-y

[32] Horowitz A Stegmann KA Riley EM. Activation of natural killer cells during microbial infections. Front Immunol. 2011;2:88. 22566877 10.3389/fimmu.2011.00088 PMC3342047

[33] Jiang L Zhang Y Li B. miRNAs derived from circulating small extracellular vesicles as diagnostic biomarkers for nasopharyngeal carcinoma. Cancer Sci. 2021;112:2393–404. 33728743 10.1111/cas.14883 PMC8177774

[34] Li Y Chen L Zhang B Ohno Y Hu H. miR-409-3p inhibits the proliferation and migration of human ovarian cancer cells by targeting Rab10. Cell Mol Biol. 2020;66:197–201. 33287942

[35] Wang Z Jin MT Yang JB. Serum miR-181c and miR-128b expression levels in patients with cerebral infarction and their correlation with 90-day prognosis. Chin J Arteriosclerosis. 2019;2706:517–21. (Chinese).

[36] Zeng L Liu J Wang Y. MicroRNA-210 as a novel blood biomarker in acute cerebral ischemia. Front Biosci. 2011;3:1265–72. 10.2741/e330 21622133

[37] Ge ZW Zhu XL Wang BC. MicroRNA-26b relieves inflammatory response and myocardial remodeling of mice with myocardial infarction by suppression of MAPK pathway through binding to PTGS2. Int J Cardiol. 2019;280:152–9. 30679074 10.1016/j.ijcard.2018.12.077

[38] Szilágyi B Fejes Z Póliska S. Reduced miR-26b expression in megakaryocytes and platelets contributes to elevated level of platelet activation status in sepsis. Int J Mol Sci. 2020;21:866. 32013235 10.3390/ijms21030866 PMC7036890

[39] Kang YC Zhang L Su Y Li Y Ren W-L Wei W-S. MicroRNA-26b regulates the microglial inflammatory response in hypoxia/ischemia and affects the development of vascular cognitive impairment. Front Cell Neurosci. 2018;12:154. 29937716 10.3389/fncel.2018.00154 PMC6002499

[40] Welch C Chen Y Stallings RL. MicroRNA-34a functions as a potential tumor suppressor by inducing apoptosis in neuroblastoma cells. Oncogene. 2007;26:5017–22. 17297439 10.1038/sj.onc.1210293

[41] Soreq H Wolf Y. NeurimmiRs: microRNAs in the neuroimmune interface. Trends Mol Med. 2011;17:548–55. 21813326 10.1016/j.molmed.2011.06.009

[42] Recchiuti A Krishnamoorthy S Fredman G Chiang N Serhan CN. MicroRNAs in resolution of acute inflammation: identification of novel resolvin D1-miRNA circuits. FASEB J. 2011;25:544–60. 20956612 10.1096/fj.10-169599 PMC3023392

[43] Fredman G Li Y Dalli J Chiang N Serhan CN. Self-limited versus delayed resolution of acute inflammation: temporal regulation of pro-resolving mediators and microRNA. Sci Rep. 2012;2:639. 22957142 10.1038/srep00639 PMC3434392

[44] Bazzoni F Rossato M Fabbri M. Induction and regulatory function of miR-9 in human monocytes and neutrophils exposed to proinflammatory signals. Proc Natl Acad Sci U S A. 2009;106:5282–7. 19289835 10.1073/pnas.0810909106 PMC2664036

[45] Ponomarev ED Veremeyko T Barteneva N. MicroRNA-124 promotes microglia quiescence and suppresses EAE by deactivating macrophages via the C/EBP-α-PU.1 pathway. Nat Med. 2011;17:64–70. 21131957 10.1038/nm.2266 PMC3044940

[46] Jones SW Watkins G Le Good N. The identification of differentially expressed microRNA in osteoarthritic tissue that modulate the production of TNF-alpha and MMP13. Osteoarthritis Cartilage. 2009;17:464–72. 19008124 10.1016/j.joca.2008.09.012

[47] Hamzei Taj S Kho W Riou A Wiedermann D Hoehn M. MiRNA-124 induces neuroprotection and functional improvement after focal cerebral ischemia. Biomaterials. 2016;91:151–65. 27031810 10.1016/j.biomaterials.2016.03.025

[48] Hertenstein T Tang Y Day AS Reynolds J Viboolmate PV Yoon JY. Rapid and sensitive detection of miRNA via light scatter-aided emulsion-based. Biosens Bioelectron. 2023;237:115444. 37329805 10.1016/j.bios.2023.115444

[49] Zhong M Chen K Sun W. PCDetection: PolyA-CRISPR/Cas12a-based miRNA detection without PAM restriction. Biosens Bioelectron. 2022;214:114497. 35797934 10.1016/j.bios.2022.114497

[50] Long G Wang F Li H. Circulating miR-30a, miR-126 and let-7b as biomarker for ischemic stroke in humans. BMC Neurol. 2013;13:1471–2377. 10.1186/1471-2377-13-178 PMC3840584 24237608

[51] Wang Y Ma Z Kan P Zhang B. The diagnostic value of serum miRNA-221-3p, miRNA-382-5p, and miRNA-4271 in ischemic stroke. J Stroke Cerebrovasc. 2017;26:1055–60. 10.1016/j.jstrokecerebrovasdis.2016.12.019 28111007

[52] Tsai PC Liao YC Wang YS Lin HF Lin RT Juo SH. Serum microRNA-21 and microRNA-221 as potential biomarkers for cerebrovascular disease. J Vasc Res. 2013;50:346–54. 23860376 10.1159/000351767

[53] Spinetti G Fortunato O Caporali A. MicroRNA-15a and microRNA-16 impair human circulating proangiogenic cell functions and are increased in the proangiogenic cells and serum of patients with critical limb ischemia. Circ Res. 2013;112:335–46. 23233752 10.1161/CIRCRESAHA.111.300418 PMC3616367

[54] Yin KJ Fan Y Hamblin M. KLF11 mediates PPARγ cerebrovascular protection in ischaemic stroke. Brain. 2013;136(Pt 4):1274–87. 23408111 10.1093/brain/awt002 PMC3613710

[55] Liu XS Chopp M Wang XL. MicroRNA-17-92 cluster mediates the proliferation and survival of neural progenitor cells after stroke. J Biol Chem. 2013;288:12478–88. 23511639 10.1074/jbc.M112.449025 PMC3642296

[56] Dhiraj DK Chrysanthou E Mallucci GR Bushell M. miRNAs-19b, -29b-2* and -339-5p show an early and sustained up-regulation in. PLoS One. 2013;8:e83717. 24376737 10.1371/journal.pone.0083717 PMC3869799

